# Trends in overweight and obesity prevalence in Tuscan schoolchildren (2002–2012)

**DOI:** 10.1017/S1368980015001676

**Published:** 2015-05-29

**Authors:** Giacomo Lazzeri, Donatella Panatto, Andrea Pammolli, Elena Azzolini, Rita Simi, Veronica Meoni, Mariano V Giacchi, Daniela Amicizia, Roberto Gasparini

**Affiliations:** 1 Department of Molecular and Developmental Medicine, University of Siena, Via A. Moro 2, 53100 Siena, Italy; 2 Department of Health Sciences, University of Genoa, Genoa, Italy; 3 Local Public Health Unit 7, Siena, Italia

**Keywords:** Overweight and obesity trend, 8–9-year-old children, BMI, Nutritional status

## Abstract

**Objective:**

The aim of the present study was to examine the prevalence and time trends in childhood overweight including obesity and obesity among Tuscan children from 2002 to 2012.

**Design:**

Cross-sectional study at five time points (Tuscan Nutritional Surveillance Surveys conducted in the years of 2002, 2006, 2008, 2010 and 2012). Trained personnel directly measured the height and weight of the subjects. BMI was assessed by means of the International Obesity Task Force (IOTF) and WHO cut-offs.

**Setting:**

Representative sample of children in the Tuscany region (Italy).

**Subjects:**

Children (*n* 7183) aged between 7·5 and 9·5 years (3711 boys and 3472 girls).

**Results:**

With respect to the estimation of the absolute prevalence level of childhood overweight, a discrepancy was observed between the two criteria. In all surveys, more boys than girls were overweight (including obesity). Trend analysis showed a significant decrease in the prevalence of overweight including obesity and obesity in Tuscan children from 2002 to 2012 (32·0 % *v*. 25·8 %, *P*<0·001 on using IOTF criteria and 37·7 % *v*. 34·3 %, *P*<0·001 on using WHO criteria for overweight including obesity; and 10·0 % *v*. 6·7 %, *P*<0·001 on using IOTF criteria and 12·5 % *v*. 11·3 %, *P*=0·035 on using WHO criteria for obesity).

**Conclusions:**

The present study is the first report from an Italian region showing a significant decrease in childhood obesity and overweight in the last 10 years. This reduction is probably a result of regional and local actions that have taken place in many sectors of society. However, efforts should be made to lower the prevalence of childhood obesity and overweight further.

Increases in the prevalence of childhood overweight (OW) and obesity (OB) have become a global public health concern^(^ ^1^ ^–^ ^3^ ^)^. Indeed, childhood OB has been recognised as an epidemic in most developed and developing countries^(^ ^4^ ^)^. Moreover, the prevalence of OW and OB among children aged 6–9 years in twelve European countries participating in the WHO European Childhood Obesity Surveillance Initiative indicates that up to 49 % of boys and 43 % of girls are overweight and up to 27 % of boys and 17 % of girls are obese (WHO growth reference)^(^ ^5^ ^)^. It is now evident from many prospective studies that childhood OB is also associated with excess weight in adulthood^(^ ^6^ ^)^. Childhood OB has also been found to be associated with the development of metabolic syndrome, diabetes and CVD in later life^(^ ^7^ ^)^. Moreover, OB in early life influences the social and psychological functioning of children^(^ ^8^ ^)^. Although increases in childhood OW have been documented in many countries around the world^(^ ^9^ ^–^ ^11^ ^)^, several recent reports have noted that the prevalence of the phenomenon has slowed and plateaued over the last 5 to 10 years in Australia^(^ ^12^ ^)^, England^(^ ^13^ ^)^, France^(^ ^14^ ^,^ ^15^ ^)^ and Sweden^(^ ^16^ ^)^.

Italy is a European country with a high prevalence of obese and overweight children, and a geographical trend has been described whereby childhood OW and OB are more prevalent in southern regions of the country than in central or northern regions^(^ ^17^ ^)^. In 2012, the prevalence rates of OW and OB in Italian children aged 9 years were reported as 22·1 % and 10·2 %, respectively. The highest values were recorded in Campania, a southern region of Italy, where 27·2 % of children were overweight and 21·5 % obese. In Tuscany, a central Italian region, the prevalence rates of OW and OB were 19·6 % and 6·9 %, respectively (International Obesity Task Force (IOTF) growth reference)^(^ ^18^ ^)^. A study estimating the prevalence of childhood OW and OB among 9-year-old children in Italy showed that the prevalence remained constant between 2008 and 2012^(^ ^19^ ^)^.

In epidemiological studies, BMI is a valid, convenient and reproducible method of assessing weight status (whereby weight becomes a surrogate for fatness). We decided to use the two main BMI cut-offs (IOTF and WHO) to classify childhood nutritional status, in order to allow comparison of our results with those of other studies. Furthermore, although these definitions sometimes yield different prevalence rates, both cut-offs have been used for international comparisons. When a sufficient number of published studies reporting prevalence values obtained by applying both cut-offs is available, algorithms can be generated which are able to estimate one cut-off from the others.

The objectives of the present study were to examine the prevalence and the time trends in childhood overweight including obesity (henceforth referred to as OWO) and OB according to two different criteria in representative samples of children in Tuscany.

## Methods

### Study subjects

We obtained data from five waves of the Tuscan Regional Nutritional Surveillance System Survey, a cross-sectional survey carried out by the Research Center for Health Education and Promotion (CREPS) of the University of Siena in 2002, 2006, 2008, 2010 and 2012. The study population consisted of children in the third grade of all public and private primary schools; almost all were 8 or 9 years old. Through the support and assistance of the Ministry of Education, we obtained lists of schools and classes, with the number of children in each class, from regional school authorities. Cluster sampling was performed according to the WHO cluster survey methodology^(^ ^20^ ^)^, with classes as the units of sampling. Identical protocols, including target group, sampling and data collection, were used in all surveys. Systematic cluster sampling (schools), stratified by administrative district, was applied in each of the five waves from which the sample was drawn. The number of children to be studied was calculated on the basis of an expected prevalence of OWO of 30 %, a desired precision of 1 % using a 95 % confidence interval, and a design effect of 2 (i.e. the number was doubled, as required by cluster sampling). A total of 7183 boys and girls aged between 7·5 and 9·5 years (1485 in 2002, 1405 in 2006, 1375 in 2008, 1424 in 2010, 1494 in 2012) participated in the Tuscan survey^(^ ^21^ ^,^ ^22^ ^)^. All five surveys had an average response rate higher than 92 %.

### Anthropometric measurements

The surveys were conducted by staff of the local health units. Each local health unit was provided with new Seca 872 scales and with Seca 214 (Seca, Hamburg, Germany) stadiometers for use during the survey. Only trained personnel measured the children’s height and weight, using appropriate, standardized instruments. For the survey of anthropometric measurements, the protocol followed the recommendations of the WHO^(^ ^23^ ^)^. Every child was weighed under standard conditions: without shoes and wearing only underclothes. We used electronic scales with a liquid crystal display that measured weight down to 100 g. Height was measured by means of a fixed or portable stadiometer with a precision of 0·1 cm; exact decimal age was calculated from the date of birth. More information on this protocol is available elsewhere^(^ ^18^ ^)^.

### Criteria for childhood overweight and obesity

BMI is used internationally to classify childhood OW and OB. Currently, the main criteria for childhood OW and OB are based on BMI information^(^ ^24^ ^–^ ^27^ ^)^. The two different criteria suggested by the IOTF and WHO present different age-specific cut-offs for childhood OW and OB^(^ ^24^ ^–^ ^26^ ^,^ ^28^ ^)^. IOTF cut-offs are extrapolated from the adult BMI cut-off points for overweight (25 kg/m^2^) and obesity (30 kg/m^2^)^(^ ^24^ ^,^ ^28^ ^)^. The WHO system defines OW as BMI >1 sd and OB as BMI >2 sd from the mean of the WHO reference population^(^ ^26^ ^,^ ^27^ ^)^.

**Fig. 1:**
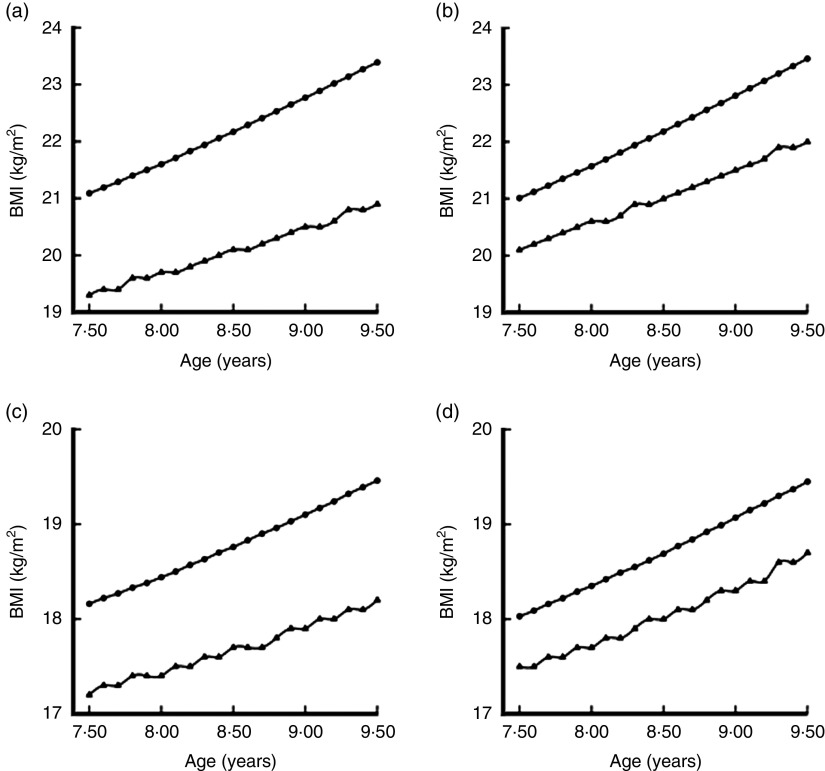
Comparison of cut-offs for childhood overweight (OW) and obesity (OB) by sex, using the criteria proposed by the International Obesity Task Force^(24,28)^ (●) and the WHO^(26,27)^ (▲), among representative samples of children aged 7·5–9·5 years, Tuscany region, Italy: (a) boys, OB; (b) girls, OB; (c) boys, OW; (d) girls, OW

Figure 1 depicts a reconstruction of the curves showing our data according to the WHO and IOTF criteria. On the basis of Fig. 1 it can be expected that, if the prevalence of childhood OB were calculated for broad age bands, the IOTF criteria would record lower levels in both boys and girls, while the prevalences recorded by means of the WHO criteria would be similar in boys and girls but greater than those yielded by the IOTF criteria^(^ ^24^ ^,^ ^28^ ^)^. The IOTF criteria for childhood OB had higher cut-offs across all ages between 7·5 and 9·5 years in both boys and girls (Fig. 1(a) and (b)). Figure 1 also shows the age-specific cut-offs for childhood OW according to the two criteria (Fig. 1(c) and (d)). For ages 7·5–9·5 years, the WHO criteria for childhood OW had lower cut-offs than the IOTF criteria among both boys and girls.

### Statistical analysis

Boys and girls were analysed separately. Continuous variables are expressed as means and standard deviations, and categorical variables are expressed as numbers of cases and percentages. The normal distribution of variables was tested by means of the Kolmogorov–Smirnov statistic. We analysed the changes in age-adjusted body weight, height and BMI and present *P* values for time trends by using linear regression analysis. To compare the means of multiple groups, we used univariate ANOVA with the *F* statistic or Welch correction for heteroscedasticity.

Levene’s test was used to test the homogeneity of variance. For multiple comparisons, we used Bonferroni’s test or Games–Howell’s test. To analyse changes in the age- and height-adjusted prevalences and 95 % confidence intervals of childhood OW and OB, we used logistic regression and estimated odds ratios and 95 % confidence intervals, while to analyse the linear trend we used the Cochran–Armitage trend test.

All analyses were conducted using the statistical software package IBM SPSS Statistics 20·0 and adjusted for cluster sampling by means of the ‘Complex Samples’ option and by using the school as the primary unit of sampling and stratifying by the school district.

## Results

Table 1 shows the number of study subjects by survey year and sex. A total of 7183 individuals (3711 boys and 3472 girls) participated in five waves; the trends in least-square mean (sd) values for body weight, height and BMI are reported. Increases in body weight were evident only among boys (*P*=0·012). Height showed statistically significant increases in both boys and girls (*P*<0·001). A significant decreasing trend in BMI was found between 2002 and 2012 in both boys and girls (*P*=0·002 and *P*=0·001, respectively).

**Table 1:** Number of study subjects and time trends in mean body weight, height and BMI among representative samples of children aged 7·5–9·5 years, Tuscany region, Italy

	2002		2006		2008		2010		2012		
	Mean	sd	Mean	sd	Mean	sd	Mean	sd	Mean	sd	*P* for trend† (2002 to 2012)
Number of subjects											
Boys (*N* 3711)	776		713		734		741		747		
Girls (*N* 3472)	709		692		641		683		747		
Body weight (kg)											
Boys	32·4	7·2	32·7	6·8	33·2	6·9	33·1	7·4	33·2	6·9	0·012
Girls	31·6	7·0	31·9	6·5	32·2	6·8	32·2	6·8	31·8	6·5	0·394
All	32·0^A^	7·1	32·3	6·7	32·7^A^	6·9	32·7	7·2	32·5	6·7	0·02
Height (cm)											
Boys	133·5^a,b^	5·9	133·6^c,d^	5·6	134·6^a,c^	5·9	134·3	6·0	135·1^b,d^	6·0	<0·001
Girls	132·5^a,b,c,d^	6·1	132·6^e,f,g^	5·9	133·6^a,e^	6·3	133·8^b,f^	6·1	133·6^d,g^	6·0	<0·001
All	133·0^a,b,c^	6·0	133·1^d,e,f^	5·8	134·1^a,d^	6·1	134·1^b,e^	6·1	134·4^c,f^	6·1	<0·001
BMI (kg/m^2^)											
Boys	18·0	3·1	18·2	2·9	17·9	2·9	17·9	3·0	17·8	2·8	0·002
Girls	17·9^a^	3·1	18·0^b,c^	2·9	17·6	2·9	17·6^b^	2·8	17·5^a,c^	2·8	<0·001
All	18·0^A^	3·1	18·1^B,C^	2·9	17·8	2·9	17·7^B^	2·9	17·6^A,C^	2·8	<0·001

^A,B,C^Only the mean values with the same superscript letters, in the same row, were significantly different (*P*<0·05; Games-Howell’s test, with correction of Welch for heteroscedasticity (Levene’s test) on four time points). ^a,b,c,d,e,f,g^Only the mean values with the same superscript letters, in the same row, were significantly different (*P*<0·05; Bonferroni’s *post hoc* test).

† Linear regression model with *F* test on four time points.

Table 2 shows the trends in the prevalence (95 % CI) of childhood OWO and OB among Tuscan boys and girls. In both boys and girls, the IOTF criteria yielded a lower prevalence of OWO and OB than the WHO criteria. For example, the prevalence of OB among boys in 2008 according to the WHO criteria (15·5 %) was nearly twice as high as that yielded by the IOFT criteria (8·4 %); similar differences were obtained for boys in both 2012 (WHO: 13·8 %; IOTF: 7·6 %) and 2006 (WHO: 18·3 %; IOTF: 10·1 %). Despite the different prevalence levels yielded by the different criteria, the time trends were similar. No significant trends in the prevalence of either OWO or OB were noticed among boys according to the WHO criteria. Both methods revealed a significant decreasing trend in the prevalence of both OWO and OB from 2002 to 2012 on considering all subjects (boys and girls together).

**Table 2:** Time trends in the prevalence of overweight including obesity (OWO) and obesity (OB) by sex and overall, using the criteria proposed by the International Obesity Task Force (IOTF)^(24,28)^ and the WHO^(26,27)^, among representative samples of children aged 7·5–9·5 years, Tuscany region, Italy

	2002		2006		2008		2010		2012		
	Prevalence	95 % CI	Prevalence	95 % CI	Prevalence	95 % CI	Prevalence	95 % CI	Prevalence	95 % CI	*P* for trend† (2002 to 2012)
Boys											
OB											
IOTF	10·3	8·2, 12·4	10·1	7·9, 12·3	8·4	6·4, 10·5	8·0	6·0, 9·9	7·6	5·7, 9·5	0·023
WHO	13·5	11·1, 15·9	18·3	15·4, 21·1	15·5	12·9, 18·2	14·6	12·1, 17·2	13·8	11·3, 16·3	0·50
OWO											
IOTF	32·6	29·3, 35·9	35·2	31·7, 38·7	29·8	26·4, 33·1	29·8	26·5, 33·3	27·4	24·2, 30·6	0·004
WHO	37·7	34·3, 41·1	45·6	41·9, 49·2	39·1	35·6, 42·7	37·7	34·2, 41·2	36·6	33·1, 40·0	0·09
Girls											
OB											
IOTF	9·7	7·5, 11·9	9·8	7·6, 12·0	6·2	4·4, 8·1	6·3	4·5, 8·1	5·7	4·1, 7·4	<0·001
WHO	11·4	9·1, 13·8	13·9	11·3, 16·4	8·2	6·1, 10·4	10·4	8·1, 12·7	8·8	6·8, 10·9	0·02
OWO											
IOTF	31·4	28·0, 34·9	36·8	33·2, 40·4	29·2	25·6, 32·7	27·1	23·7, 30·4	24·1	21·0, 27·2	<0·001
WHO	37·8	34·2, 41·4	41·9	38·2, 45·6	35·7	32·0, 39·4	34·0	30·4, 37·5	32·1	28·8, 35·5	0·001
All											
OB											
IOTF	10·0	8·5, 11·6	10·0	8·4, 11·5	7·4	6·0, 8·8	7·2	5·8, 8·5	6·7	5·4, 8·0	<0·001
WHO	12·5	10·8, 14·2	16·1	14·2, 18·0	12·1	10·4, 13·9	12·6	10·8, 14·3	11·3	9·7, 12·9	0·035
OWO											
IOTF	32·0	29·7, 34·4	36·0	33·5, 38·5	29·5	27·1, 31·9	28·5	26·2, 30·8	25·8	23·5, 28·0	<0·001
WHO	37·7	35·2, 40·2	43·7	41·1, 46·3	37·5	35·0, 40·1	35·9	33·4, 38·4	34·3	31·9, 36·7	<0·001

† Using the Cochran–Armitage test.

Table 3 shows age- and height-adjusted OR (95 % CI) of OWO and OB among boys and girls.

**Table 3:** Age- and height-adjusted odds ratios (and 95 % confidence intervals) of overweight including obesity (OWO) and obesity (OB) by sex and overall (reference year 2002), using the criteria proposed by the International Obesity Task Force (IOTF)^(24,28)^ and the WHO^(26,27)^, among representative samples of children aged 7·5–9·5 years, Tuscany region, Italy

		2006		2008		2010		2012	
	2002	OR	95 % CI	OR	95 % CI	OR	95 % CI	OR	95 % CI
Boys									
OB									
IOTF	1·00	0·86	0·60, 1·22	0·78	0·54, 1·13	0·75	0·51, 1·08	0·64**	0·44, 0·93
WHO	1·00	1·34	1·00, 1·80	1·15	0·85, 1·55	1·08	0·80, 1·47	0·94	0·69, 1·27
OWO									
IOTF	1·00	1·04	0·82, 1·30	0·84	0·66, 1·06	0·86	0·68, 1·08	0·69**	0·54, 0·87
WHO	1·00	1·35**	1·09, 1·68	1·00	0·81, 1·25	0·96	0·77, 1·20	0·85	0·68, 1·06
Girls									
OB									
IOTF	1·00	0·84	0·58, 1·22	0·67	0·44, 1·03	0·66	0·44, 1·00	0·63**	0·42, 0·95
WHO	1·00	1·15	0·83, 1·60	0·70	0·48, 1·01	0·88	0·62, 1·25	0·76	0·53, 1·08
OWO									
IOTF	1·00	1·16	0·92, 1·46	0·89	0·70, 1·15	0·78	0·61, 1·00	0·69**	0·54, 0·88
WHO	1·00	1·11	0·89, 1·39	0·90	0·71, 1·13	0·81	0·64, 1·02	0·76**	0·61, 0·96
All									
OB									
IOTF	1·00	0·85	0·66, 1·10	0·73**	0·56, 0·97	0·70**	0·53, 0·92	0·64**	0·48, 0·84
WHO	1·00	1·25**	1·01, 1·55	0·94	0·75, 1·19	0·98	0·78, 1·24	0·85	0·68, 1·08
OWO									
IOTF	1·00	1·10	0·93, 1·29	0·86	0·73, 1·02	0·82**	0·69, 0·97	0·69**	0·58, 0·82
WHO	1·00	1·23**	1·05, 1·43	0·95	0·81, 1·12	0·89	0·76, 1·04	0·81**	0·69, 0·95

**P<0·01.

## Discussion

We evaluated the trend in the prevalence of childhood OWO and OB estimated on the basis of two different criteria: WHO and IOTF. With regard to the estimation of the absolute prevalence of childhood OWO, a discrepancy between the two criteria was observed. These findings indicate that, when reporting the prevalence of childhood OW and OB, it is crucial to clarify the reference criterion in order to avoid any potential confusion. However, it is not possible, within the scope of the present study, to suggest the ‘best’ criterion for monitoring childhood OW and OB, given that the ‘best’ criterion should be selected according to its ability to predict future health outcomes. Therefore, investigations into the health outcomes associated with these different cut-off points are needed^(^ ^29^ ^)^. Despite reports of alarming international trends in childhood OW and OB^(^ ^6^ ^,^ ^14^ ^)^, our study found no further rise in the prevalence of OWO between 2006 and 2012 in children aged 8–9 years in Tuscany. A significant decrease in OWO and in OB was observed after 2002 (*P* for trend <0·001). Although the two criteria yielded differences in the absolute prevalence of OW and OB, they did not display differences in the time trends of the prevalence. This means that either criterion can be legitimately used in determining the time trends in childhood OW and OB. In Italy, only one other study has evaluated the prevalence of OW and OB among 9-year-old children, showing constant prevalence rates between 2008 and 2012. The authors used the IOTF cut-offs as BMI criteria^(^ ^19^ ^)^. Moreover, a geographical trend has been described, whereby childhood OW and OB are more prevalent in southern Italy than in central or northern Italy^(^ ^18^ ^)^. Another study by Parrino *et al*. also reported significantly increasing OW and OB in 11–13-year-old boys and girls over the last decade in Sicily (a southern Italian region)^(^ ^29^ ^)^ on using the IOTF cut-offs.

The phenomenon observed in our study (decrease in OWO and OB) has also been registered by international surveillance, which has shown a steady or a decreasing trend of OW in children in recent years in the USA^(^ ^30^ ^)^, Ireland^(^ ^31^ ^)^ and Argentina^(^ ^32^ ^)^. In particular, Lioret *et al*.^(^ ^14^ ^)^ reported that the prevalence of OWO, as assessed by means of IOTF BMI criteria, is currently stabilizing among French children aged 3–14 years. In a study of Danish infants, children and adolescents (1998–2011), Schmidt *et al*. discerned a levelling-off in the prevalence of OW and OB (IOTF growth reference)^(^ ^33^ ^)^. Ebbeling and Ludwig suggested that, in recent years, public health campaigns aimed at raising awareness of childhood OB and improving the quality of food in schools could explain the levelling-off of trends in the USA^(^ ^30^ ^)^. Stamatakis and colleagues indicated that extensive media attention on OB and the consequent increase in the awareness of this problem at the individual and family level, as well as public anti-obesity policies, might have helped to blunt the rise in OB^(^ ^13^ ^)^.

Although there have been several reports on the prevalence of childhood OB, our study is important mostly because of its strengths in terms of high compliance (92 %) and strictness in methodology and data collection. Furthermore, we utilized the two most commonly used criteria for estimating childhood OW and OB in order to favour the comparison of our results with those of other studies and to provide other researchers with the opportunity to compare their data with ours, something which has rarely been attempted in previous Italian studies. In addition, we used several rounds of representative anthropometric data, while taking into account primary sampling units and stratification and also examining recent trends in childhood OW and OB.

Like all nutritional status studies, the present study has limitations. The use of BMI as a measure of weight status has been criticized, especially in children, because BMI may be affected by skeletal structure and muscle mass^(^ ^34^ ^)^. However, BMI in this age group is highly correlated with body fat mass and BMI is considered appropriate for monitoring and comparing prevalence at the population level^(^ ^35^ ^,^ ^36^ ^)^. Using only data from prevalence based on BMI cut-off criteria (generating categories of weight status) does not capture changes over time based on BMI distribution.

Finally, the present paper is the first report from an Italian region to show a significant decrease in childhood OWO and OB in the last 10 years. The decrease in OWO and OB is even more important if we consider that childhood OB correlates with OB later in life^(^ ^6^ ^,^ ^37^ ^,^ ^38^ ^)^ and with morbidity in adulthood^(^ ^39^ ^)^. Over 30 % of Italian adults are overweight and about 8 % are obese, meaning that approximately 15 million adults are overweight and 4 million obese^(^ ^40^ ^)^. Excess weight (including the conditions of OW and OB) is the sixth greatest risk factor for the ‘global burden of diseases’ and is associated with several non-communicable diseases, including CVD such as hypertension and stroke, diabetes, cirrhosis, osteoarthritis and sleep apnoea^(^ ^41^ ^)^. In Italy, non-communicable diseases are estimated to account for 92 % of all deaths^(^ ^42^ ^)^.

The decrease in the prevalence of childhood OWO and OB registered in the current cross-sectional study may be explained by successful public health initiatives. These may have contributed to this levelling-off of OWO and OB trends. Since 2002, the Tuscany region has fostered a tradition of public health policies, official nutrition recommendations, dietary guidelines and collaboration with schools^(^ ^43^ ^)^, which has probably laid a solid foundation for the recent initiatives. Indeed, many local activities in child health care have been started in pre-schools and schools since 2000. More recently (2006) these activities have been incorporated into regional health-care plans and have led to the institution and consolidation of activities concerning nutritional surveillance and health-promotion projects. For instance, the book *Forchetta e Scarpetta* and the DVD entitled *Open Mind*, edited by the project ‘OKKIO alla Salute’^(^ ^18^ ^)^, have been used to promote a healthy lifestyle among children, and specific programmes to encourage children’s consumption of fruit and vegetables, such as ‘…. e vai con la frutta’^(^ ^44^ ^)^, have been implemented. Nevertheless, the actual impact of these initiatives needs to be examined in future evidence-based studies.

## Conclusions

In conclusion, the data collected on children aged between 7·5 and 9·5 years in Tuscany (Central Italy) from 2002 to 2012 show a significant decrease in the prevalence of OWO and OB. However, continuous future monitoring of OWO in children is important in order to ascertain whether the decline revealed in the present study is only a temporary phenomenon or a sign that the epidemic is in fact reversing. In order to bring childhood OWO rates down to acceptable levels and to reach less affluent groups, concerted, multi-sectoral, long-term actions are needed, in combination with much greater political determination.
